# Impact of offering cycle training in schools upon cycling behaviour: a natural experimental study

**DOI:** 10.1186/s12966-016-0356-z

**Published:** 2016-03-08

**Authors:** Anna Goodman, Esther M. F. van Sluijs, David Ogilvie

**Affiliations:** Faculty of Epidemiology and Population Health, London School of Hygiene and Tropical Medicine, Keppel Street, London, WC1E 7HT UK; MRC Epidemiology Unit and UKCRC Centre for Diet and Activity Research (CEDAR), University of Cambridge School of Clinical Medicine, Box 285, Cambridge Biomedical Campus, Cambridge, CB2 0QQ UK

**Keywords:** Cycling, Cycle training, Children, Natural experiment, Bikeability

## Abstract

**Background:**

England’s national cycle training scheme, ‘Bikeability’, aims to give children in England the confidence to cycle more. There is, however, little evidence on the effectiveness of cycle training in achieving this. We therefore examined whether delivering Bikeability was associated with cycling frequency or with independent cycling.

**Methods:**

We conducted a natural experimental study using information on children aged 10–11 years participating in the nationally-representative Millennium Cohort Study. We identified Cohort participants whose schools had offered Bikeability in 2011–2012 using operational Bikeability delivery data (children in London excluded, as delivery data not available). Our natural experimental design capitalised on the fact that Cohort participants were surveyed at different times during 2012 and were also offered Bikeability at different times during 2012. This allowed us to compare cycling levels between children whose schools delivered Bikeability before their survey interview (‘intervention group’, *N* = 2563) and an otherwise comparable group of children whose schools delivered Bikeability later in the year (‘control group’, *N* = 773). Parents reported whether their child had completed formal cycle training; their child’s cycling frequency; whether their child ever made local cycling trips without an adult; and other child and family factors. We used Poisson regression with robust standard errors to examine whether cycling behaviour differed between the intervention and control groups.

**Results:**

Children whose school had offered Bikeability were much more likely to have completed cycle training than the control group (68 % vs. 28 %, *p* < 0.001). There was, however, no evidence that delivering Bikeability in school was associated with cycling more often (49.0 % cycling at least once per week in the intervention group vs. 49.6 % in the control group; adjusted risk ratio 0.99, 95 % CI 0.89, 1.10). There was likewise no evidence of an association with cycling independently (51.5 % in the intervention group vs. 50.1 % in the control group; adjusted risk ratio 0.97, 95 % CI 0.89, 1.06).

**Conclusions:**

Offering high-quality cycle training free at the point of delivery in English schools encourages children to do cycle training, but we found no evidence of short-term effects on cycling frequency or independent cycling. Future evaluation should investigate longer-term effects on these and other stated Bikeability objectives such as increasing cycling safety.

**Electronic supplementary material:**

The online version of this article (doi:10.1186/s12966-016-0356-z) contains supplementary material, which is available to authorized users.

## Background

Increasing the proportion of journeys made by bicycle would be expected to confer important transport, health and environmental benefits [1–4], and has increasingly become a policy priority in many countries over the past decade [5–8]. Providing a supportive physical environment is likely to be one key component of encouraging people to cycle [9, 10], and this may be particularly successful if complemented by programmes seeking to boost motivation, confidence or cycling skills [11, 12]. For this reason, many cycling promotion strategies combine ‘hard’ environmental engineering measures with ‘soft’ measures such as personalised travel planning or cycle training (e.g. [13, 14]).

In England, one flagship ‘soft’ policy measure has been the introduction of the Bikeability cycle training scheme, which was launched by the Department for Transport in 2007. Aiming to provide “cycling proficiency for the 21st century”, Bikeability offers high-quality cycle training designed for children in the final years of primary school [15, 16]. The primary, most proximate goal of the Bikeability scheme is to give children the skills and confidence to cycle safely on the road [15, 16]. It is hoped that this, in turn, will help realise the additional, ultimate policy goals of reducing cycling injuries and increasing cycling frequency – Bikeability was originally conceived as part of a broader strategy to get “more people cycling, more safely, more often” [15].

In relation to the goal of increasing cycling frequency, Bikeability is potentially important because parental and child concerns related to traffic safety are among the key determinants of children’s travel and play behaviours, including with respect to cycling [17, 18]. By boosting children’s cycling skills and confidence, Bikeability is thought to encourage both children and their parents to see cycling as a viable mode of transport, and therefore to get children to use their bicycles more often [15, 16]. An alternative or additional possible outcome would be to increase the proportion of children permitted to make cycle trips without an adult. Such an outcome might help explain any increase in overall cycling levels (e.g. some children would cycle more because they were now allowed to cycle without their parents) or might occur without overall cycling levels changing (e.g. some children who previously cycled to school escorted by their parents would now cycle to school with friends). In either case, such an increase would go some way towards reversing the dramatic decline in the ‘independent mobility’ of 10–11 year olds that has been observed since the 1970s, and could help boost children’s (and parents’) freedom to organise their lives in ways of their own choosing [19, 20]. In this respect, it is potentially significant that Bikeability was typically delivered via schools to whole groups of children: given how much of children’s ‘independent’ travel in fact occurs with peers [21, 22], it is plausible that some cycling trips might only be undertaken if both a child and the child’s friends had completed cycle training. One might therefore expect the combined effects of delivering cycle training to an entire school year group to be larger than the sum of the effects of delivering training to just a few individuals in that year [22].

Yet although it is certainly the case that cycle training is widespread in high-cycling settings like Denmark and the Netherlands [23], relatively little direct evidence exists as to whether such training does in fact encourage cycling. Most studies of cycle training have focussed on impacts on knowledge, skills, safety behaviour or accident rates, with reviews providing evidence of some positive effects for the first three outcomes but null or inconclusive findings for the latter (with interpretation often complicated by low statistical power) ([24, 25] see also, [26, 27]). This focus on knowledge and skills also characterises the most robust previous evaluation of Bikeability, which reports improvements in hazard perception and confidence levels, but which did not examine impacts on cycling frequency [28].

Among those studies that have examined effects of cycling training on cycling frequency, a survey of 1974 British children in 1994/95 reported mixed results. Specifically, there was little difference between trained and untrained children in total cycling levels as judged by a 1-week ‘cycling log book’, but 60 % of trained children said in a questionnaire that they cycled on the road more often after they had been trained [29]. A cluster randomised controlled trial in Belgium in 2012 found no effect of a cycle training programme on the prevalence of cycling to school 5 months later, but the small sample size (*N* = 3 schools, 94 children) means that this may simply reflect low statistical power [26]. In relation specifically to changes in cycling frequency following Bikeability training, a cross-sectional ecological study has reported that the local authorities implementing cycle training are also the local authorities with the highest proportion of secondary school children cycling to school [16]. While certainly encouraging, interpretation of these findings is complicated by the substantial potential for selection bias or confounding: it could simply be that cycle training is implemented in places that already have high cycling rates or that are simultaneously rolling out other cycling initiatives. One further ‘proof of concept’ research study of Bikeability is similarly encouraging but inconclusive. This study found higher levels of cycling among 68 trained children than among 156 untrained children, but it is unclear how far this reflects the fact that most of the untrained group were a year younger than the trained group [30].

This paper therefore capitalised upon an opportunity to use a large, nationally-representative cohort study to conduct a more robust evaluation of this flagship government scheme. Specifically, we aimed to use data from the Millennium Cohort Study to examine whether Bikeability cycle training was associated with (i) an increased frequency of cycling, (ii) a greater likelihood of ever going on cycle trips without an adult (‘cycling independently’), and (iii) a greater likelihood of cycling to school. We also aimed to examine whether the impact of Bikeability cycle training differed across different subgroups of children. In doing so, we sought to generate evidence with direct policy relevance for the Department for Transport. We also sought to contribute to the wider international evidence base as to which cycling initiatives are effective and how their effectiveness may vary between groups.

## Methods

### Intervention: Bikeability cycle training

The Bikeability cycle training scheme aims to give children “practical skills and understanding [about] how to cycle on today’s roads” during the final years of primary school [31]. The content of the training is underpinned by the National Standard for cycle training, and includes both off-road training (Level 1) and on-road training (Level 2). Level 1 training develops children’s bicycle handling skills in an off-road environment, e.g. starting and stopping with control, changing gears and looking behind. Level 2 training takes place on-road, and covers skills needed to make short journeys on local roads, e.g. knowing where to ride on the road, passing parked cars and navigating simple junctions. There also exists a Bikeability Level 3 module that delivers more advanced on-road training (e.g. navigating complex junctions and roundabouts), but this is rarely delivered in primary schools.

The large majority of Level 1 and Level 2 Bikeability training is delivered via schools during school hours, and most children complete both levels in the course of around 4 sessions lasting 2 h each. The training is delivered by instructors who are either directly employed by the local authority or who work for a third party organisation that is under contract to the local authority. All instructors must be trained to the National Standard for cycle training and registered with a Bikeability scheme accredited by the Department for Transport. Children are asked to bring their own bikes for the training, although children who do not own a roadworthy bike may be loaned a ‘pool’ bike. Parents are asked for their permission for their child to take part.

Since its launch in England in 2007, coverage of the Bikeability scheme has increased every year. By 2011/2012 around 55 % of all schools in England (52 % of schools covered in the Millennium Cohort Study) offered the training [32], and around half of all children in England take part in Bikeability [16]. Schools can offer Bikeability training free of charge, with costs covered by central and local government funding; in 2011/2012 the estimated cost to central government was £11 million (~16 million USD/14.5 million Euros) [16]. In November 2015, the government committed a further £50 million to maintain the scheme up to 2020 [33].

### Sample

**Fig. 1:**
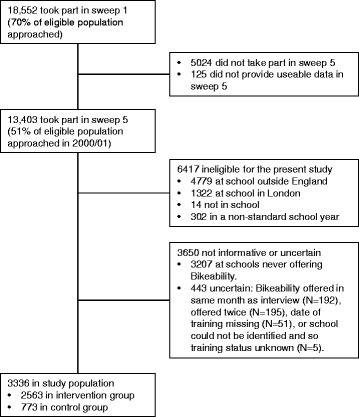
Children participating in the Millennium Cohort Study, and their selection into our study population for analysis

The Millennium Cohort Study (MCS) is a nationally-representative sample of British children that has been characterised in detail across five sweeps [34, 35]. The first sweep took place in 2001/02 when the children were around 9 months old; subsequent sweeps have happened in 2003/04, 2006, 2008 and 2012. The data available draw on extended interviews with parents, some direct measurements (e.g. of height and weight), plus briefer interviews with teachers and children in the more recent sweeps. The fifth sweep (Year 6, age 10–11) successfully collected data on 13,403 children (51 % of those eligible to participate in the first MCS sweep). Of these children, we excluded 6417 who were not eligible for our analyses and 3650 whose data were not informative for our pre-specified comparisons (see Fig. 1, plus next section for more details). The resulting study population therefore comprised 3336 children, sampled between January 2012 and August 2012.

Parents of children participating in MCS provided informed, written consent and their children provided oral assent. Ethical approval for the fifth sweep of the MCS was granted by the Yorkshire and Humber research ethics committee (Ref:11/YH/0203 [35]). Ethical approval for our analyses was granted by the London School of Hygiene and Tropical Medicine ethics committee (Ref: 7034).

### Primary exposure groups for controlled comparisons: school-level Bikeability delivery

**Fig. 2:**
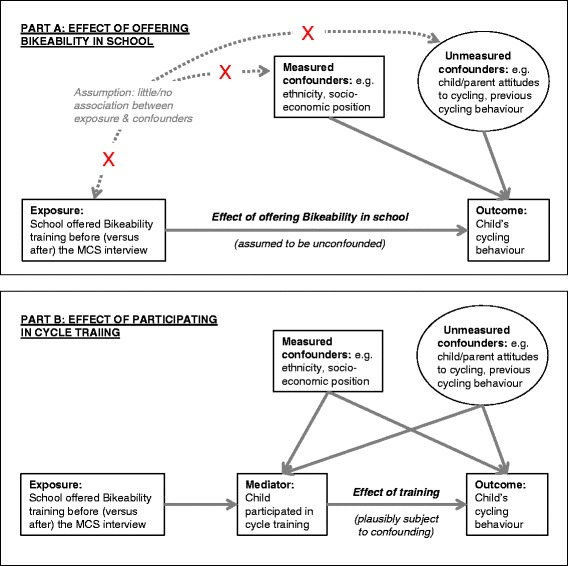
Conceptual models guiding analyses. MCS = Millennium Cohort Study. In this model, square boxes denote measured variables; circles denote unmeasured variables; and solid lines show hypothesised causal relationships. Part A shows the conceptual model guiding our primary analyses, concerning the effect of offering Bikeability in school upon cycling frequency. Part B shows a more detailed version of this conceptual model that hypothesises the role of participation in cycle training as a mediator, and also shows how participation in cycle training might be subject to confounding by measured and unmeasured characteristics

We have previously shown that children who received cycle training differed systematically from those who did not (e.g. in being more likely to play sports), and that schools that offered Bikeability differed systematically from schools which did not (e.g. in containing a somewhat more affluent student body) [32]. In order to minimise the potential bias introduced by such confounders, we pursued a ‘natural experimental’ [36] approach predicated upon the fact that schools offer the training at different points throughout the school year, and MCS participants were also interviewed at different points in the year. Specifically, we decided a priori to make our primary comparison between children whose school offered Bikeability training prior to the date of the survey interview versus children whose school offered the training later on in the same year. We adopted this approach on the assumption, which we subsequently confirmed empirically, that these two groups would be similar in their individual, family and school characteristics (assumption depicted in Fig. 2, Part A). We therefore believed that the comparison of these groups would be less subject to residual confounding, and would provide a relatively unbiased estimate of the effect on cycling frequency of offering Bikeability training in school. Moreover, this school-level comparison is also arguably particularly relevant for policy, given that encouraging more schools to offer Bikeability is the most straightforward way that central government could seek to increase the impact of the scheme, We recognise that this comparison will, however, tend to underestimate any effect of actually *completing* Bikeability, insofar as not all children who are offered the cycle training take part, and not all of those who take part successfully complete the Level 2 training.

To define the ‘intervention’ and ‘control’ groups, we used operational data provided by the Department for Transport to identify schools that offered Bikeability to children in the year group covered by the Millennium Cohort Study. This involved identifying schools that offered Bikeability to Year 5 children (age 9–10) in the academic year 2010/11 or to Year 6 children (age 10–11) in the academic year 2011/12 (see Additional file 1 for further details). Data on schools in London were not available. The available Bikeability delivery data, including the month in which training took place, were merged with the MCS data using the Unique Reference Number of each child’s current school. This allowed us to identify children whose school offered Bikeability training (1) before the date of the survey interview, either in Year 5 or earlier in Year 6 (‘intervention group’) or (2) after the date of the survey interview, later in Year 6 (‘control group’). As a sensitivity analysis, we also examined whether the impacts of Bikeability might vary according to how recently the training took place. We did this by examining whether there was any difference within the intervention group according to whether Bikeability training had been offered 1–5 months prior to the interview versus 6 months or more before.

### Outcome data: children’s cycling behaviour

Our primary outcome was the frequency with which each child cycled. To measure this, we administered to parents an item based on the UK National Travel Survey [37], asking “How often does [Child] use a bicycle? Please include travel to and from school”. The response categories were: every day or almost every day; several times a week; once or twice a week; at least once a month; every few months; at least once a year; less often or never. This question was purposively commissioned for inclusion in MCS in collaboration with the Department for Transport, as was the question on cycle training participation described below. Reliability and validity data for these questions are not available. Our secondary outcomes were whether the child usually travelled to school by bicycle; and whether, excluding the journey to school, the child ever made bicycle trips around the local area without an adult. Again, both of these were measured by parental report.

### Mediator: child-level completion of cycle training

During the MCS interview, parents were asked “Has [Child] ever done any formal cycling proficiency training such as ‘Bikeability’? Formal cycling proficiency training is delivered by a recognised trainer and includes tuition on the road” (response categories yes/no). This provided our measure of whether the child had participated in cycle training, which we hypothesised to mediate any association between the school offering Bikeability and the child’s cycling behaviour (Fig. 2, Part B). This variable also provided an alternative, individual-level measure of exposure to cycle training, albeit one which we believed likely to be importantly confounded by both measured and unmeasured characteristics (Fig. 2, Part B). As such, although we considered this variable to offer some scope for estimating the effect of actually participating in cycle training upon cycling frequency, we also recognised the possibility that an estimated association would be subject to unmeasured confounding.

### Potential confounders

The child, family and area confounders that we considered are presented in Table 1. Data on child and family characteristics were almost all provided in the fifth sweep of MCS. The only exception was whether the child cycled to or from school at age 7, which was provided in the fourth sweep and which is the only measure of the child’s cycling prior to the fifth sweep. All child and family characteristics relied on parental report except for weight status, which was derived using measures of height and weight taken by trained interviewers during the survey interview of the fifth sweep of MCS, and which was defined using standard cut-points [38]. The local prevalence of cycling was defined as the proportion of adult commuters who cycled to work in the 2011 Census [39], a measure which provides a reasonable proxy for the prevalence of cycling in general in an area [40]. This measure was used because correspondingly fine-grained measures of cycling participation among children are not available, and because adult modal share and child modal share are fairly highly correlated at a regional level (Pearson correlation coefficient 0.72) [41]. The local prevalence of cycling was merged with the MCS database according to the Lower Super Output Area of the child’s home address (Lower Super Output Areas are administrative areas containing around 1500 individuals). The home Lower Super Output Area was also matched to the 2004 Rural and Urban Area Classification [42], and used to define settlement type (large urban areas with a population >10,000; smaller towns and fringe areas; and villages, hamlets and isolated dwellings).

**Table 1:** Characteristics of study population (*N* = 3336)

Variable	Level	Full study population (*N* = 3336)	Control group (*N* = 773)	Intervention group (*N* = 2563)	*P*-value for difference
Sex	Female	1667 (50 %)	366 (47 %)	1301 (51 %)	0.10 ^c^
	Male	1669 (50 %)	407 (53 %)	1262 (49 %)	
Age	10 years	1212 (36 %)	364 (47 %)	848 (33 %)	<0.001 ^c^
	11 years	2124 (64 %)	409 (53 %)	1715 (67 %)	
Ethnicity	White	2859 (86 %)	646 (84 %)	2213 (86 %)	0.22 ^c^
	Mixed	104 (3 %)	26 (3 %)	78 (3 %)	
	South Asian	306 (9 %)	87 (11 %)	219 (9 %)	
	Black	41 (1 %)	8 (1 %)	33 (1 %)	
	Other	26 (1 %)	6 (1 %)	20 (1 %)	
Weight status	Normal/underweight	2376 (74 %)	552 (74 %)	1824 (74 %)	0.97 ^d^
	Overweight	652 (20 %)	156 (21 %)	496 (20 %)	
	Obese	197 (6 %)	43 (6 %)	154 (6 %)	
General health	Good/excellent	3245 (97 %)	753 (98 %)	2492 (97 %)	0.64 ^c^
	Fair/poor	90 (3 %)	19 (2 %)	71 (3 %)	
Longstanding illness	No	2864 (86 %)	662 (86 %)	2202 (86 %)	0.93 ^c^
	Yes	468 (14 %)	109 (14 %)	359 (14 %)	
Frequency of attending club or classes for sport or other exercise	Not at all	844 (25 %)	189 (24 %)	655 (26 %)	0.23 ^d^
	At most once a week	770 (23 %)	168 (22 %)	602 (23 %)	
	2–3 times a week	1187 (36 %)	286 (37 %)	901 (35 %)	
	4–5 times a week	535 (16 %)	130 (17 %)	405 (16 %)	
Cycled to/from school age 7	No	3027 (99 %)	703 (99 %)	2324 (99 %)	0.52 ^c^
	Yes	36 (1 %)	10 (1 %)	26 (1 %)	
Highest education of either parent^a^	Degree	516 (15 %)	106 (14 %)	410 (16 %)	0.50 ^d^
	Diploma	1234 (37 %)	320 (41 %)	914 (36 %)	
	Higher secondary	499 (15 %)	114 (15 %)	385 (15 %)	
	Middle secondary	685 (21 %)	140 (18 %)	545 (21 %)	
	Low, other or none	397 (12 %)	93 (12 %)	304 (12 %)	
Equivalised household income^b^	Fifth 1 (highest)	667 (20 %)	167 (22 %)	500 (20 %)	0.44 ^d^
	Fifth 2	749 (22 %)	182 (24 %)	567 (22 %)	
	Fifth 3	714 (21 %)	149 (19 %)	565 (22 %)	
	Fifth 4	638 (19 %)	128 (17 %)	510 (20 %)	
	Fifth 5 (lowest)	568 (17 %)	147 (19 %)	421 (16 %)	
Highest occupational social class of either parent	High managerial/professional	490 (15 %)	124 (16 %)	366 (14 %)	0.80 ^c^
	Low manager/professional	934 (28 %)	209 (27 %)	725 (29 %)	
	Intermediate	453 (14 %)	101 (13 %)	352 (14 %)	
	Small employers & self-employed	304 (9 %)	66 (9 %)	238 (9 %)	
	Low supervisory & technical roles	126 (4 %)	25 (3 %)	101 (4 %)	
	Semi-routine	291 (9 %)	69 (9 %)	222 (9 %)	
	Routine	177 (5 %)	38 (5 %)	139 (5 %)	
	Not economically active	529 (16 %)	130 (17 %)	399 (16 %)	
Settlement type	Large urban area	2745 (82 %)	624 (81 %)	2121 (83 %)	0.28 ^c^
	Small town & fringe	263 (8 %)	63 (8 %)	200 (8 %)	
	Village or smaller	323 (10 %)	86 (11 %)	237 (9 %)	
Prevalence of cycling to work in local area	<2 %	1603 (48 %)	401 (52 %)	1202 (47 %)	0.01 ^d^
	2–3.9 %	1141 (34 %)	249 (32 %)	892 (35 %)	
	4–5.9 %	354 (11 %)	83 (11 %)	271 (11 %)	
	≥6 %	238 (7 %)	40 (5 %)	198 (8 %)	

^a^ Includes both academic and vocational qualifications. ‘Degree’ corresponds to British National Vocational Qualification (NVQ) level 1, ‘Diploma’ to NVQ2, ‘Higher secondary’ to NVQ3, ‘Middle Secondary’ to NVQ2 and ‘Low, other or none’ to NVQ1, overseas qualifications or no qualifications

^b^ Equivalised for household composition in terms of adults and children [35]

^c^ Chi-squared test for association

^d^ Chi-squared test for trend

### Statistical analysis

After first presenting descriptive analyses, we fit regression models in which the outcomes were (i) cycling at least once a week, (ii) ever cycling, (iii) cycling to school and (iv) ever making non-school bike trips in the local area without an adult. For these regression analyses we used Poisson regression with robust standard errors [43]; unlike logistic or ordered logistic regression, this provides an estimate of the risk ratio for common outcomes. These regression analyses allowed for the stratified sampling design used in the first sweep of MCS; allowed for clustering of children within schools (*N* = 1444 unique schools for the 3336 participants); and used the fifth-sweep MCS weights assigned to each child to allow for differences in sampling rates, response rates and follow-up rates. The models adjusted for the characteristics presented in Table 1, plus the region in England where the child lived and the season of data collection (January-March, April-May and June-August).

The proportion of missing data was 3 % for weight status, 8 % for whether the child cycled to school at age 7, and <0.2 % for all other variables presented in Table 1. Missing data were imputed using multiple imputation by chained equations (five imputations) under an assumption of missing at random. We tested a priori for interactions between the child’s exposure to Bikeability and (i) the child’s sex, (ii) parental education and income, (iii) settlement type or (iv) the prevalence of cycling in the local area. Statistical analyses were conducted using Stata 13.1.

Following the primary evaluation (i.e. comparing children offered Bikeability in school with children not offered Bikeability in school), we re-ran the analyses comparing children who had participated in cycle training with those who had not. We additionally calculated the expected percentage-point difference between the intervention and control groups under the assumption that any differences observed with respect to cycle training status reflected a fully causal role of training upon cycling levels (see Additional file 1 for equations). We compared this expected difference to the effect actually observed in the primary evaluation. If the expected effect was larger than the observed effect, this suggested that differences observed between trained and untrained children reflected residual confounding rather than a causal role of cycle training.

## Results

### Comparability of intervention and control groups

In total, 2563 children in our sample were at schools that had already offered cycle training at the time of the survey (a median of 8 months previously) and 773 children were at schools that would offer cycle training later in the year (a median of 3 months subsequently). As shown in Table 1, the characteristics of these ‘intervention’ and ‘control’ groups were generally very similar. The only significant differences were that the local prevalence of adult commuter cycling was slightly higher for the intervention group and that the intervention group was older (this latter difference is unsurprising, since children interviewed later in the year were progressively more likely to have already been offered Bikeability). These results therefore provided some evidence to support the assumption depicted in Fig. 2 Part A, namely that our primary comparison groups were broadly similar.

Yet although very similar in most respects, the intervention and control groups did differ markedly in the proportion of children reported by their parents to have received cycle training. This proportion was 68 % (95 % CI 66, 70 %) in the intervention group as opposed to 28 % (25, 32 %) in the control group. This confirmed our assumption that being offered Bikeability in school would predict participating in cycle training, as depicted in Fig. 2 Part B.

### Association between school offering Bikeability and cycling behaviour

**Fig. 3:**
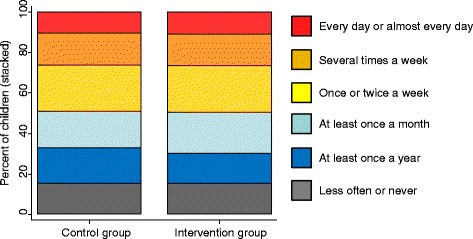
Children’s frequency of cycling, according to whether their school had already offered them Bikeability (‘intervention group’) or offered it later in the year (‘control group’). Response categories ‘every few months’ and ‘at least once a year’ combined, because only 3 % of parents selected the latter

As shown in Fig. 3, the frequency of cycling was very similar between our intervention and control groups – that is, between children whose schools had offered cycle training and those whose schools had not offered training (chi-squared *p* = 0.50 for association, *p* = 0.54 for trend). Likewise in regression analyses adjusting for child, family and area characteristics, there was no evidence of an association between whether the school had offered Bikeability and whether the child (i) cycled at least once a week, or (ii) ever cycled (Table 2). There was also no evidence that the intervention and control groups differed in terms of whether the child usually cycled to school, or ever made local bicycle trips independently. In addition, there was no evidence of any difference within the intervention group between those who had been offered Bikeability training 1–5 months prior to the interview and those who had been offered the training 6 months or more before (all *p* > 0.15). For no outcome was there evidence of differential effects between the intervention and control group with respect to the child’s sex, the parent’s education or income level, the settlement type or the prevalence of cycling in the local area (all *p* > 0.05 for interaction, most *p* > 0.2).

**Table 2:** Associations between whether the school offered Bikeability and children’s cycling behaviour across the study population (*N* = 3336)

Outcome	Exposure group	Percentage (95 % CI)	Unadjusted analysis (risk ratio, 95 % CI)	Adjusted analysis (risk ratio, 95 % CI)
Child cycles at least once a week	Control group	49.0 (45.4, 52.6)	1	1
	Intervention group	49.6 (47.6, 51.5)	1.02 (0.92, 1.13)	0.99 (0.89, 1.10)
Child ever cycles	Control group	84.7 (82.0, 87.2)	1	1
	Intervention group	84.5 (83.1, 85.9)	0.99 (0.95, 1.03)	0.99 (0.95, 1.04)
Child usually travels to school by bike	Control group	2.8 (1.8, 4.3)	1	1
	Intervention group	2.8 (2.2, 3.5)	0.98 (0.57, 1.69)	0.73 (0.41, 1.29)
Child makes local bike trips independently^a^	Control group	50.1 (46.5, 53.6)	1	1
	Intervention group	51.5 (49.5, 53.5)	1.00 (0.91, 1.09)	0.97 (0.89, 1.06)

All *p* ≥ 0.4 for association. Analyses based on our study population of 3336 children, of whom 773 were in the control group and 2563 in the intervention group. Adjusted analyses adjusted for all variables shown in Table 1 (with the local prevalence of cycling to work entered as a continuous variable), and also for the region of England that the child lived in and the season of data collection

*CI* confidence interval

^a^ Defined as ever making local, non-school bicycle trips without an adult, either on their own or with other children

### Association between participation in cycle training and cycling behaviour

By contrast, whether the child had participated in cycle training was strongly associated with cycling more often and with being more likely to cycle independently (Table 3). For example, the prevalence of cycling at least weekly was 55 % among children who had received cycle training and 41 % among children who had not. If this association were fully causal, the expected prevalence of cycling weekly would be (0.55 * 0.68) + (0.41 * (1–0.68)) = 51 % among children in the intervention group whose schools offered Bikeability (the value 0.68 corresponds to the 68 % of children in those schools who had received cycle training). The corresponding expected prevalence in the control group would be (0.55 * 0.28) + (0.41 * (1–0.28)) = 45 %. That we did not even observe a trend towards such a 6-percentage-point difference suggests that the association between participation in cycling training and cycling behaviour is largely or entirely non-causal, but is instead accounted for by residual or unmeasured confounding.

**Table 3:** Associations between previous cycle training and children’s cycling behaviour across the study population (*N* = 3336)

Outcome	Whether child had done cycle training	Percentage (95 % CI)	Unadjusted analysis (risk ratio, 95 % CI)	Adjusted analysis (risk ratio, 95 % CI)
Child cycles at least once a week	Untrained	41.5 (38.9, 44.2)	1	1
	Trained	55.0 (52.8, 57.2)	1.27 (1.17, 1.39)	1.26 (1.16, 1.37)
Child ever cycles	Untrained	73.0 (70.6, 75.4)	1	1
	Trained	92.7 (91.4, 93.8)	1.23 (1.18, 1.28)	1.20 (1.15, 1.25)
Child usually travels to school by bike	Untrained	1.9 (1.2, 2.8)	1	1
	Trained	3.4 (2.7, 4.3)	1.62 (0.99, 2.67)	1.38 (0.83, 2.29)
Child makes local bike trips independently^a^	Untrained	43.3 (40.6, 45.9)	1	1
	Trained	56.7 (54.5, 58.9)	1.23 (1.13, 1.34)	1.21 (1.11, 1.32)

All *p* < 0.001 for association. Analyses based on our study population of 3336 children, of whom 1378 were untrained, 1956 trained and 2 had missing data (imputed using multiple imputation). Adjusted analyses adjusted for all variables shown in Table 1 (with the local prevalence of cycling to work entered as a continuous variable), and also for the region of England that the child lived in and the season of data collection

*CI* confidence interval

^a^ Defined as ever making local, non-school bicycle trips without an adult, either on their own or with other children

## Discussion

Bikeability is a national cycling training programme reaching around half of children in England in their final years of primary school. In this observational, natural experimental study of 3336 English 10–11 year olds, we found no evidence that offering Bikeability in school had a short-term effect on cycling frequency in children. There was similarly no evidence that children who had been offered Bikeability in school were more likely to cycle independently of an adult, or of differential effects according to the child’s sex, socio-economic background, settlement type or whether they lived in a high-cycling area.

### Strengths and limitations

As a large, established, nationally-representative birth cohort, the Millennium Cohort Study (MCS) provides a powerful resource for investigating a broad range of topics of interest to policy-makers. Nevertheless, our study is comparatively innovative in having harnessed these strengths to inform a specific, current government initiative. In doing so, we were able to generate high-quality evidence that could otherwise only have been obtained at considerably greater expense. We hope that other researchers may consider using similar approaches in the future. Another important strength of this study lies in its identification of intervention and control groups that differed in their likelihood of having completed cycle training but that appeared broadly similar with respect to other child, family and area characteristics. This provided us with a more robust basis for controlled comparisons than most previous evaluations in this field, including previous evaluations of Bikeability [16, 30].

One key limitation of this study is the relatively short period of follow-up; as we discuss below in more detail, it is possible that the effects of cycle training may not emerge until children progress to secondary school, i.e. after the period of our survey. In addition, although our use of MCS is what made this study possible to conduct at all, limited questionnaire space meant that it was only possible for us to commission two parent-reported cycling questions in the MCS interview. Ideally, our two measures of cycling frequency and independent cycling would have been complemented by more detailed questions covering different types of cycling (e.g. on- versus off-road); by complementary questions to the children; and perhaps by a more detailed measurement technique such as a cycling log book or activity diary. The absence of such information may have introduced some measurement error with respect to our outcome measures, although we do not expect any differential measurement bias between the two groups and therefore expect the impact on our conclusions to be limited.

Another important limitation is that we could not conduct a more comprehensive examination of the impacts of Bikeability. The reflected our lack of data on other major outcomes of interest, most notably child and parental perceptions of children’s skills and confidence levels, and cycling-related injuries. For a fuller understanding of the relationships explored in this paper, it would also have been informative to have access to other variables that might confound, moderator or mediate any effects of cycle training on cycling frequency, such as parents’ or children’s attitudes towards cycling, or subjective or objective measures of the quality of the local cycling environment. We recommend that this should be considered in future research.

### Meaning of the study and directions for further research

Although we found no evidence that offering Bikeability in school was associated with cycling behaviour, we did find strong evidence of a positive association between a child having completed formal cycle training and that child’s cycling frequency. Taken together, these findings suggest that the association between cycle training participation and cycling behaviour is not causal, but instead reflects cycle training being sought out by parents and children who already cycled or who intended to start cycling. How one interprets this finding perhaps depends on what one sees as the main purpose of cycle training. Insofar as one primary aim of cycle training is to improve cycling skills and cycling safety, then children who are already cycling are arguably those who need it most. By contrast, to the extent that cycle training is additionally intended to give children the confidence to cycle more often or to take up cycling, then priority should also be given to current non-cyclists. Under either interpretation, from a methodological perspective our finding highlights the potential danger of simply comparing ‘trained’ and ‘untrained’ children [29, 30], suggesting that such comparisons may be vulnerable to a bias analogous to ‘confounding by indication’ [36].

As for our null finding regarding the effect of offering cycle training in schools on cycling behaviour, this replicates two previous studies that have asked about past week cycling behaviour [26, 29]. In interpreting this finding, it is telling that around half of the children in our sample were reported to cycle every week but under 3 % usually cycled to school. This suggests that most of the reported cycling may have been recreational, and hints at the scale of the challenge facing those who are seeking to encourage a wider range of types of cycling in this age group. In this context, it seems plausible that cycle training may not be enough to effect change unless it is delivered as part of a broader, multifaceted intervention [26]. In the past year the Department for Transport has started to pilot a more intensive scheme ‘Bikeability Plus’ that combines cycle training with other measures, such as facilitating access to second-hand bicycles or organising joint cycle rides for parents and children. In line with some evidence from school-based interventions targeting overall physical activity [44, 45], it is possible that the impact of this more intensive programme will be enhanced by its multi-component format and its efforts to include parents. Then again, our null finding should also be placed in the context of the strong preference that children and parents place upon low-traffic or traffic-free cycle routes [46, 47]. This raises the possibility that cycle training might only increase children’s cycling frequency in the context of a sufficiently supportive physical cycling environment. If this is true, then the potential benefits of Bikeability might not be realised unless combined with a broader package of environmental improvement measures, as has been done in some of England’s ‘Cycling Towns’ [13, 48].

Bikeability alone may therefore not be enough to increase children’s cycling in the context of the current cycling environment in England. Yet on the other hand, changes in cycling levels occur within a complex system [49], and we have shown in one previous study of cycling initiatives that effects only became apparent in the medium term [50]. As such, it is also possible that effects of offering Bikeability training will emerge as children get older and progress to secondary school, especially given that this is a period when many parents start allowing children greater freedom to cycle alone and to cycle on the road [17]. Such a delayed effect would be consistent with previous reports that cycling among secondary school children has increased in local authorities that have delivered more Bikeability training relative to local authorities that have delivered less [16].

In conclusion, this study provided no evidence that offering Bikeability in schools affected how often children cycled in the short term, but was unable to examine potential effects on cycling safety or on cycling frequency in the longer term. We therefore intend to re-examine the effects of Bikeability upon the Millennium Cohort Study participants three years later, including through using the detailed time-use diaries that will be collected at age 14. We also hope that this more extended follow-up of the effects of Bikeability cycle training will be complemented by other research into impacts on skills and safety, and by a parallel evaluation of the more intensive proposed program ‘Bikeability Plus’. Given our demonstration that there is substantial potential for residual confounding when comparing ‘trained’ and ‘untrained’ children, all such future studies should choose the most robust evaluation designs possible. Through such evaluations we hope that it will be possible to build an evidence base as to how schemes like Bikeability affect cycling, and over what time scale.

## Additional file

Additional file 1: Supplementary methods information. This provides further details as to 1) the nature of the operational Bikeability data and 2) the equation used to calculate expected differences in the intervention and control group based on observed differences between trained and untrained children. (DOC 110 kb)

## Abbreviations

CI: confidence intervals
MCS: Millennium Cohort Study
NVQ: National Vocational Qualification
