# Supplementary material for: Impact of offering cycle training in schools upon cycling behaviour: a natural experimental study
Source: Int J Behav Nutr Phys Act. 2016 Mar 8;13:34. doi: 10.1186/s12966-016-0356-z (PMC4784314; doi:10.1186/s12966-016-0356-z)
Supplement: Additional file 1: — Supplementary methods information. This provides further details as to 1) the nature of the operational Bikeability data and 2) the equation used to calculate expected differences in the intervention and control group based on observed differences between trained and untrained children. (DOC 110 kb) [file 12966_2016_356_MOESM1_ESM.doc]

**Additional Material 1: Supplementary methods to “Impact of offering cycle training in schools upon cycling behaviour: a natural experimental study”**

**Bikeability delivery data**

We sought to identify all schools that had offered Bikeability cycle training to the cohort of children leaving primary school in 2012, using operational data provided by the Department for Transport. Schools are encouraged to deliver Bikeability in the final year of primary school (Year 6, age 10-11), but a minority instead deliver the training a year earlier (Year 5, age 9-10). We therefore sought to identify all schools that offered Bikeability either to Year 6 children in the academic year 2011/12 or else to Year 5 children in the academic year 2010/11. This corresponds to the cohort of children who left primary school in 2012, which is the year group covered by the Millennium Cohort Study.

Unfortunately, the Department for Transport collects some delivery data using financial years (which run from April to March) rather than academic years (which run from September to August), and the Department did not routinely collect data on the month of delivery until April 2011. This meant that for a proportion of Year 5 delivery, we could identify schools that had delivered Bikeability between April 2010 and March 2011, but could not tell if the delivery had occurred between April 2010 and August 2010 (i.e. too soon to affect our MCS cohort) or between September 2010 and March 2011 (i.e. in the right academic year to affect our MCS cohort).

Throughout the analyses presented in this paper we assumed that these ambiguous schools did in fact offer Bikeability to our cohort members – i.e. our definition of which schools offered Bikeability was somewhat over-inclusive. Few schools were, however, affected by this decision. For example, removing these ambiguous schools reduced the size of our intervention group from 2563 children to 2262 children. All our findings were very similar in a sensitivity analysis that excluded these children. Similarly all our analyses were unchanged in a sensitivity analyses that excluded all children whose schools offered Bikeability in Year 5, and that only compared children according to whether they had yet been offered Bikeability in Year 6.

Finally, we should note that uncertainty regarding the date of Bikeability delivery in some 2010/11 data is not the only potential source of measurement error. The operational delivery data was compiled from a range of sources and providers, and some did not always provide data in the format in which it was requested. As a result, the organisation compiling the Bikeability delivery data has advised that some delivery data prior to 2013/14 “may not be 100 per cent accurate” (see http://bikeability.org.uk/download/494/). Having said this, the strong correlation that we observed between the operational data (‘whether the school had offered Bikeability’) and the MCS data (‘parental report as to whether their child had completed cycle training’) gives us reason to believe that this potential measurement error did not compromise the validity of the primary comparison in this paper.

**Calculation of expected difference between intervention and control groups, based on the observed difference between trained and untrained children**

Our primary comparison was between our ‘intervention’ and ‘control groups’, i.e. between children who had been offered Bikeability in school at the time of the Millennium Cohort survey and children who had not yet been offered Bikeability. We additionally compared children who had participated in cycle training with those who had not. We next calculated the expected percentage-point difference between the intervention and control groups under the assumption that any differences observed between trained and untrained children reflected a fully causal role of training upon cycling levels. We did this using the following equations:

%Cycle_IntSchool = [%Cycle_Trained * %Trained_IntSchool] + [%Cycle_Untrained * (1 – %Trained_IntSchool))

%Cycle_ContSchool = [%Cycle_Trained * %Trained_ContSchool] + [%Cycle_Untrained * (1 – %Trained_ContSchool))

Expected percentage-point difference = %Cycle_IntSchool - %Cycle_ContSchool

In these equations, “%Cycle_IntSchool” indicates the expected percentage of children cycling in intervention schools, and “%Cycle_ContSchool” indicates the equivalent in control schools; “Cycle_Trained” indicates the observed percentage of children cycling among those who have received cycle training, and “Cycle_Untrained” is the equivalent among children who have not received training; and “%Trained_IntSchool” indicates the observed percentage of children in intervention schools who have participated in cycle training, and “%Trained_ContSchool” is the equivalent in control schools.

To the extent that the expected difference between the intervention and control group was larger than the difference actually observed, this suggested that any difference between trained and untrained children reflected residual confounding rather than a causal role of the cycle training.
